# Necrotizing Fasciitis and Toxic Shock Syndrome from *Clostridium septicum* following a Term Cesarean Delivery

**DOI:** 10.1155/2014/724302

**Published:** 2014-04-13

**Authors:** B. H. Rimawi, W. Graybill, J. Y. Pierce, M. Kohler, E. A. Eriksson, M. T. Shary, B. Crookes, D. E. Soper

**Affiliations:** ^1^Division of Reproductive Infectious Diseases, Department of Obstetrics and Gynecology, Medical University of South Carolina, 96 Jonathan Lucas Street, Charleston, SC 29425, USA; ^2^Department of Obstetrics and Gynecology, Medical University of South Carolina, 96 Jonathan Lucas Street, Charleston, SC 29425, USA; ^3^Division of Gynecological Oncology, Department of Obstetrics and Gynecology, Medical University of South Carolina, 96 Jonathan Lucas Street, Charleston, SC 29425, USA; ^4^Division of Trauma Surgery and Critical Care, Department of General Surgery, Medical University of South Carolina, 96 Jonathan Lucas Street, Charleston, SC 29425, USA

## Abstract

Necrotizing fasciitis and toxic shock syndrome are life-threatening conditions that can be seen after any surgical procedure. With only 4 previous published case reports in the obstetrics and gynecology literature of these two conditions occurring secondary to *Clostridium septicum*, we describe a case of necrotizing fasciitis and toxic shock syndrome occurring after a term cesarean delivery caused by this microorganism, requiring aggressive medical and surgical intervention.

## 1. Introduction

Each year, more than 1 million cesarean deliveries are performed in the United States [1]. Rates of surgical site infection after cesarean delivery have dramatically declined with the use of antibiotic prophylaxis, but postoperative wound infection remains a significant consequence. While treatment of postoperative infections most commonly involves opening the incision, a serious infection may require more aggressive surgical intervention. Necrotizing fasciitis is the most serious consequence of abdominal wound infection. The microbiology of wound infections resulting in necrotizing fasciitis is primarily polymicrobial; however, these pathogens can be highly virulent due to the elaboration of potent virulence factors. Clostridial infection is an example of such a virulent pathogen. Clostridial infections, most commonly* Clostridium perfringens*, have the ability to release neurotoxic exotoxins and histotoxins, leading to gas gangrene, widespread necrotizing soft tissue infection, and death.* Clostridium septicum* accounts for only a small number of clostridial infections but carries a high morbidity and mortality risk [2]. We describe a patient who survived a widespread necrotizing soft-tissue infection and toxic shock syndrome due to* Clostridium septicum* following a cesarean delivery.

## 2. Case Report

A 23-year-old primigravid with no known past medical history had an uncomplicated prenatal course until she began having clinical signs of preeclampsia at 39-week gestation. All prenatal labs were unremarkable and her antenatal culture for group B streptococcus was negative. Her body mass index was 23 kg/m^2^. Physical examination including respiratory, abdominal, and cardiac was unremarkable, except for pitting edema in her lower extremities. Initial laboratory tests including a complete blood count, hepatic panel, basic metabolic panel, coagulation profile, and fibrinogen level were within normal limits.

She was started on intravaginal misoprostol for induction of labor, and was noted to have spontaneous rupture of amniotic membranes with clear, non-foul smelling amniotic fluid several hours after labor induction. She progressed to full cervical dilation; however, a protracted labor course despite effective maternal pushing was noted. She had 7 vaginal examinations for cervical assessment performed after the time of induction, of which 3 examinations being performed after rupture of her amniotic membranes. Total duration of labor was 28 hours, with 24 hours in the first stage and 4 hours in the second stage of labor.

Her cesarean delivery was uncomplicated without evidence of overt infection or bleeding. Her skin was closed with a monocryl suture in a subcuticular fashion. Her postoperative course was unremarkable and she was discharged home on the third postoperative day. At the time of discharge, her white blood cell (WBC) count was 11,200 cells/*μ*L and her hemoglobin was 11.6 gm/dL.

On postoperative day seven, four days after discharge, she complained of fever, chills, and night sweats, along with incisional pain. She was advised to come to the hospital for evaluation. Her temperature was 38.5°C, her pulse was 125 beats per minute, respiratory rate was 28 breaths per minute, and blood pressure was 110/75 mm/Hg. On physical examination, her abdomen was tender to palpation, and there were extensive surrounding erythema and induration. Her WBC count was 28,400 cells/*μ*L with 85% neutrophils and 6% bands, hemoglobin was 8.2 gm/dL, platelet count was 206,000 cells/*μ*L, serum sodium level was 132 mEq/L, serum creatinine was 1.8 mg/dL, aspartate aminotransferase (AST) was 130 IU/L, and alanine aminotransferase (ALT) was 220 IU/L. Computed tomography of her chest, abdomen, and pelvis revealed an otherwise enlarged postpartum uterus without evidence of gas gangrene or uterine hysterotomy dehiscence; however, ascites was noted, along with evidence of a pulmonary embolus.

**Figure 1:**
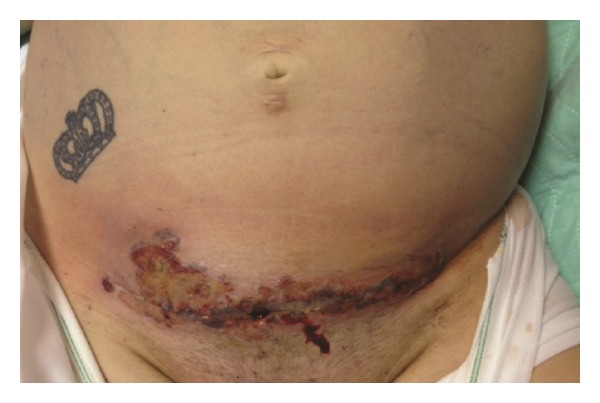
Abdominal examination on presentation to the tertiary care center on postoperative day 12.

She received fluid resuscitation, intravenous vancomycin, piperacillin-tazobactam, and was anticoagulated with unfractionated heparin. Four days later (postoperative day 11), she remained febrile. Her WBC count rose to 40,600 cells/*μ*L with 40% bands, hemoglobin was 6.8 gm/dL, platelet count was 126 cells/*μ*L, serum sodium level was 125 mEq/L, serum creatinine was 2.6 mg/dL, AST was 560 IU/L, ALT was 739 IU/L, and serum bicarbonate level was 15 mmol/L. Her wound cellulitis continued to progress with evidence of hemorrhagic skin bullae with surrounding necrosis and crepitus (Figure 1). She was transferred to the tertiary-care center on postoperative day 12, whereupon her broad-spectrum antibiotics were changed to ceftriaxone and clindamycin, a regimen we commonly use at our institution to cover the polymicrobial nature seen with most obstetrical and gynecological wound infections, and she was prepared for surgery. Multidisciplinary teams consisting of an experienced gynecological oncologist and general surgeon, as well as infectious disease specialists, were consulted.

**Figure 2:**
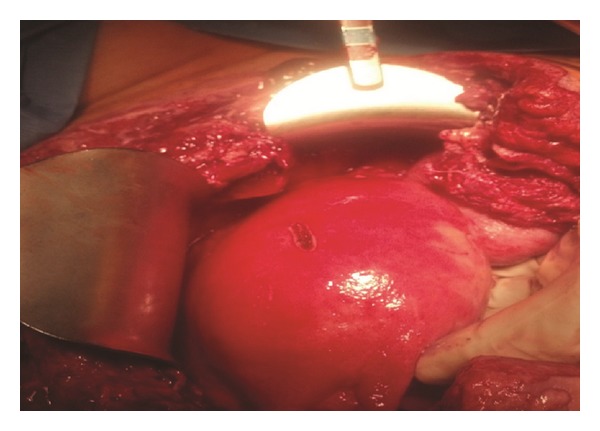
Intra-operative uterus examination demonstrating good vascularity on postoperative day number 12.

**Figure 3:**
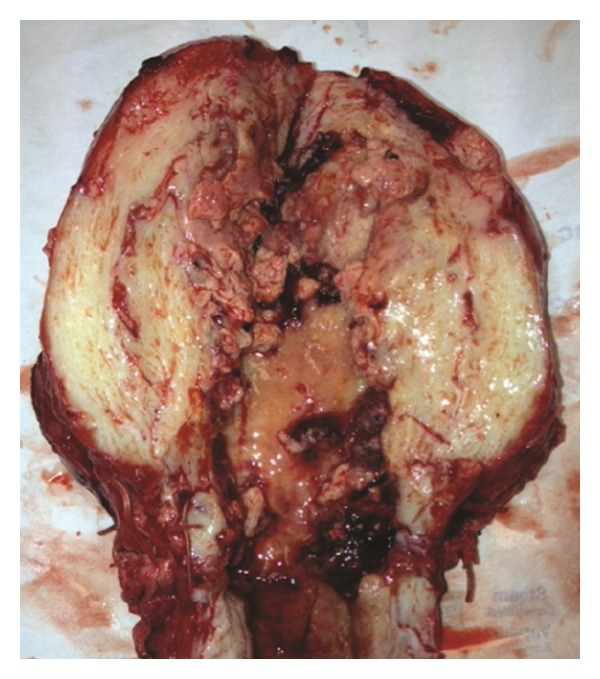
Post-operative uterus examination demonstratingextensive necrosis of the myometrium with acute inflammation, extending from the lower uterine segment to the uterine fundus.

A diagnosis of necrotizing fasciitis was made and she was taken to the operating room for debridement of her wound. After the wound was opened, evidence of greyish necrotic tissue was noted. All necrotic tissue, involving the skin, subcutaneous tissue, rectus muscles and fascia was excised. On examination of her uterus, purulent fluid drained from her hysterotomy site, which was opened, debrided to a bleeding edge, and closed. Endometrial curettage was performed through the hysterotomy prior to its closure. Good perfusion of the uterus (confirmed by noting active bleeding after incision into the anterior serosa and myometrium) and adnexa was appreciated (Figure 2). She underwent daily wound debridements and peritoneal cavity washouts and the uterus remained hyperemic in appearance. Due to persistent fevers with leukocytosis, on postcesarean section day 15, her antibiotics were changed to high dose penicillin and clindamycin in order to cover the most serious microorganisms that cause necrotizing fasciitis and toxic shock syndrome (e.g., group A strep and clostridial species). On postcesarean day 17, intraoperative examination noted a dusky-appearing uterus with absence of bleeding when an incision was made into the uterus. A total abdominal hysterectomy was therefore performed with pathology revealing extensive necrosis of the myometrium with acute inflammation, extending from the lower uterine segment to the uterine fundus (Figure 3). Additional tissue samples from her uterus and anterior abdominal wall were sent to the Center for Disease Control and Prevention (CDC) for molecular microbiologic analysis. Results of her endometrial curettage revealed granulation tissue with fibrinous debris and benign myometrium tissue with acute necrotic debris.

**Figure 4:**
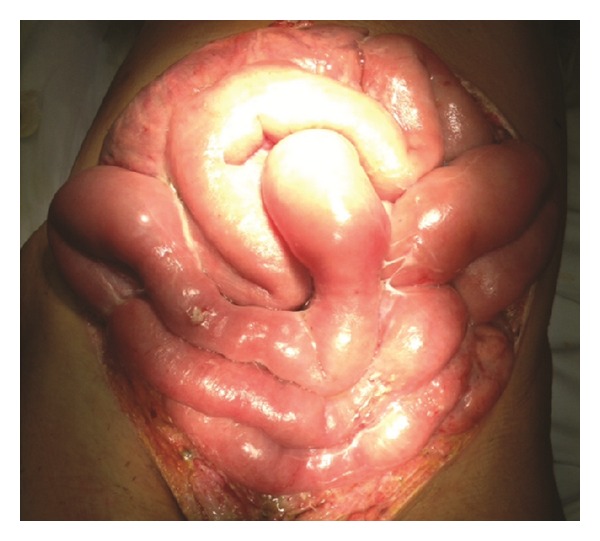
Depicts complete resection of the rectus abdominal muscles and fascia—20 × 25 cm open anterior abdominal defect on postoperative day number 20.

During each visit to the operating room, extensive debridement of her anterior abdominal wall was performed; however, during her 8th visit, postcesarean day 20, even more extensive debridement was undertaken to 2 cm beyond the bleeding edges to insure adequate resection (Figure 4). Subsequent visits to the operating room failed to show any further anterior abdominal wall necrosis with healthy appearing tissue and granulation tissue over the bowel beginning to develop. A 20 × 25 cm open anterior abdominal defect with no rectus abdominal muscles or fascia remained (Figure 4).

**Figure 5:**
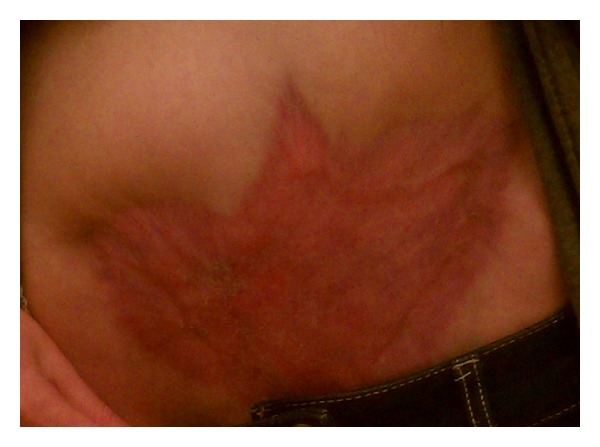
Multiple reconstructive surgeries have resulted in complete closure of her anterior abdominal wall.

The remainder of her hospital course was complicated by disseminated intravascular coagulopathy and multiorgan failure, which gradually resolved with aggressive supportive care, blood transfusions, and resuscitation over four weeks. Improvement began on postoperative day 20, once adequate margins on her anterior abdominal wall were obtained. While hysterectomy was important, it was the abdominal wall resection that ended up being the most critical. Once adequate resection was effected, her clinical condition improved. High dose penicillin and clindamycin were continued until she became afebrile. After postoperative cesarean day number 20, there was also a gradual improvement in all her labs, along with intermittent spaced-apart, low-grade fevers, rather than continuous, ranging from 37.5-38.0°C. On hospital day 51, she was discharged home. Over the next 3 months, her abdominal wall defect had successfully completely granulated and she underwent 2 additional reconstructive surgeries to close her abdominal wall defect (Figure 5).

All the samples collected during her surgeries including tissue biopsies, blood and urine cultures, Gram stains, and cytology were negative for microorganisms. Results of the tissue samples of her anterior abdominal wall and her uterus from the surgery performed on postcesarean day 17 that were sent to the CDC for molecular microbiologic analysis were available on postoperative day 41. Samples of both tissue specimens revealed no immunohistochemical evidence of clostridial species or streptococcal species, including group A or B streptococcus infection. No molecular or special stain evidence of clostridial species, including* Clostridium perfringens* and* Clostridium sordellii*, or streptococcal species were found. Using DNA extracted from the uterine tissue, the test for* Clostridium septicum* alpha toxin was detected by a specific PCR assay with 99% homology.* Prevotella bivia* DNA was also detected by broad range eubacterial 16S rDNA and sequencing.

## 3. Discussion

Necrotizing soft-tissue can involve all the layers of the soft tissue compartments (dermis, subcutaneous tissue, superficial fascia, deep fascia, and muscle) as well as deeper tissues in the pelvis (organ spaces). Such infections are infrequent but carry high morbidity and mortality. As obstetricians and gynecologists rarely encounter such infections, they can remain unrecognized, resulting in delay of surgical treatment; thereby, resulting in progressive necrosis.

We searched the English-language literature published until January 2014 in the PubMed database. Relevant studies were identified using various keyword combinations including “gynecology,” “*Clostridium septicum*,” “obstetrics,” and “necrotizing fasciitis.” No lower publication date limit was set. Four cases of* Clostridium septicum* infections in obstetrical and gynecological patients were ascertained. Two reported cases have occurred after medical abortion [1, 2] and two were involved with occult gynecological malignancies [3, 4]. The novelty of this case is that no prior report describes a patient who survived widespread necrotizing soft-tissue infection and toxic shock syndrome associated with* C. septicum* after an uncomplicated term primary cesarean delivery.

The microbiology of necrotizing fasciitis can be polymicrobial or monomicrobial, of which both can lead to rapid destruction of skin, subcutaneous tissue, fascia and/or muscle. Of these infections, clostridial and group A streptococcal myonecrosis are two of the most fulminant infections in humans.* Streptococcus pyogenes* not only leads to severe postpartum endometritis but can also cause tissue destruction which progresses rapidly into necrotizing fasciitis [5]. Of the clostridial species,* Clostridium perfringens* is the most common associated with necrotizing fasciitis [6, 7].

*Clostridium septicum* can release an alpha-toxin, a necrotizing pore-forming cytolysin that is secreted as an inactive protoxin [8]. This protoxin was isolated using a* C. septicum*-specific PCR testing in our patient. These small pore-forming toxins have a range of effects on the target cell that have lytic and vacuolating properties responsible for* C. septicum*-mediated clostridial myonecrosis and destruction of otherwise healthy tissues [8, 9].

Pertinent signs and symptoms typically include a sudden onset of severe pain and edema surrounding a surgical site with or without nonpurulent exudate. Diffuse bronzing of the skin with crepitus is often noted; however, in more advanced disease, the addition of cutaneous gangrene and/or hemorrhagic bullae is also present. Multiorgan failure and septic shock can quickly follow such devastating infections, including acute liver and renal failure, as seen with our patient. Laboratory associations with necrotizing fasciitis typically involve a leukocytosis (>25,000 cells/*μ*L), anemia (hemoglobin < 11 mg/dL), hyponatremia (<125 mEq/L), hyperglycemia (>180 mg/dL), a creatinine level of greater than 1.6 mg/dL, and a bicarbonate level below 22 mmol/L [10].

Optimal therapy of postoperative wound infection includes opening the incision to not only allow for the drainage of a possible deeper wound abscess but also to assess the tissues for possible necrosis [10]. Necrosis is characterized by tissue that is dusky, associated with a dishwater-like exudate, and does not bleed when incised. Early intervention is felt to possibly prevent further extension of infection and therefore further tissue necrosis.

In this case, the patient was taken to surgery for wound debridement promptly when she was received in transfer. The typical approach of debriding the incision to bleeding edges was undertaken. In addition, the hysterotomy incision appeared to be involved so this was debrided back to bleeding edges as well. The decision for hysterectomy in these cases is always difficult but can be life-saving. In this case, the uterine serosa was incised and prompt bleeding noted suggesting that the uterus was not necrotic and therefore was not in need of extirpation at the time of initial exploration (postoperative cesarean day number 12). Daily explorations and resections of the anterior abdominal incision allowed for routine inspection of the uterus, which finally appeared necrotic on postoperative day number 17. Hysterectomy revealed a largely necrotic uterus. Interestingly, the uterine curettage obtained at the time of initial exploration on postoperative day number 12 revealed necrotic decidua (not surprising) but also necrotic myometrium. This suggests that the* Clostridium septicum* ascended from the vagina, infecting the endomyometrium and hysterotomy site resulting in an “in-to-out” type of necrosis, which finally devitalized the uterus. One might expect an earlier necrosis from toxin elaboration, but this was not apparent when looking at the serosal side of the uterus through an abdominal incision. Incising the serosa and looking for bleeding may not be a valid way of deciding as to the advisability of hysterectomy.

Despite a change in antibiotic therapy to include high dose penicillin (postop day 15) and hysterectomy (postop day 17), the patient did not improve. Continued debridement of the anterior abdominal was performed daily, always with resection to bleeding edges. However, on postoperative day number 20, an even more extensive debridement was undertaken ensuring that a 2 cm margin was obtained from all edges. It was following this debridement that the patient's clinical condition began to improve. This observation suggests the need, at least in the case of infections involving* C. septicum*, that resection even beyond that resulting in bleeding edges is important. Once this level of resection was obtained the patient improved resulting in her ultimate discharge from the hospital on day 52.

This patient's survival was the result of a highly motivated persistent multidisciplinary team of surgeons and infectious disease consultants. The mortality rate associated with necrotizing fasciitis without surgical therapy approaches 100%. Surgical intervention lowers this mortality rate by up to 50% [11].* C. septicum* infection, even when treated surgically, is almost uniformly fatal. These necrotizing infections call for aggressive surgical debridement in combination with judicious antimicrobial therapy and intensive care multisystem support.
